# The DNA methylation inhibitor induces telomere dysfunction and apoptosis of leukemia cells that is attenuated by telomerase over-expression

**DOI:** 10.18632/oncotarget.2917

**Published:** 2015-02-17

**Authors:** Xiaolu Zhang, Bingnan Li, Nick de Jonge, Magnus Björkholm, Dawei Xu

**Affiliations:** ^1^ Department of Medicine, Division of Hematology and Center for Molecular Medicine, Karolinska Institutet and Karolinska University Hospital Solna, Stockholm, Sweden

**Keywords:** AML, Apoptosis, DNMT inhibitors, Telomerase, Telomere, TERT

## Abstract

DNA methyltransferase inhibitors (DNMTIs) such as 5-azacytidine (5-AZA) have been used for treatment of acute myeloid leukemia (AML) and other malignancies. Although inhibiting global/gene-specific DNA methylation is widely accepted as a key mechanism behind DNMTI anti-tumor activity, other mechanisms are likely involved in DNMTI's action. Because telomerase reverse transcriptase (TERT) plays key roles in cancer through telomere elongation and telomere lengthening-independent activities, and TERT has been shown to confer chemo- or radio-resistance to cancer cells, we determine whether DNMTIs affect telomere function and whether TERT/telomerase interferes with their anti-cancer efficacy. We showed that 5-AZA induced DNA damage and telomere dysfunction in AML cell lines by demonstrating the presence of 53-BP1 foci and the co-localization of 53-BP1 foci with telomere signals, respectively. Telomere dysfunction was coupled with diminished TERT expression, shorter telomere and apoptosis in 5-AZA-treated cells. However, 5-AZA treatment did not lead to changes in the methylation status of subtelomere regions. Down-regulation of TERT expression similarly occurred in primary leukemic cells derived from AML patients exposed to 5-AZA. TERT over-expression significantly attenuated 5-AZA-mediated DNA damage, telomere dysfunction and apoptosis of AML cells. Collectively, 5-AZA mediates the down-regulation of TERT expression, and induces telomere dysfunction, which consequently exerts an anti-tumor activity.

## INTRODUCTION

DNA methyltransferases (DNMTs) are responsible for the addition of methyl groups to CpG sites that are most frequently clustered in regulatory or promoter regions of genes, and appropriate DNA methylation plays important parts in controlling gene transcription. [1] Because the dysregulation of DNMT expression and aberrant DNA methylation widely occurs in human malignancies, the strategy to inhibit DNA methylation against cancer has been developed, and DNMT inhibitors (DNMTIs) including decitabine and 5-azacitidine(5-AZA) have been applied to the treatment of acute myeloid leukemia (AML), myelodysplastic syndromes (MDS), and other malignancies. [1–5] It is generally believed that DNMTis result in global and gene-specific hypomethylation through which growth arrest and/or apoptosis of malignant cells are induced. [1, 6] A typical example is the tumor suppressor p16^INK4^, a gene that is frequently silent due to its promoter methylation in oncogenesis while re-activated by DNMTI treatment. [7] However, DNMTI-mediated growth arrest and apoptosis of leukemic cells has recently been shown to result from the generation of reactive oxygen species (ROS). [8] In addition, activation-induced cytidine deaminase was down-regulated by DNMTIs via proteasomal degradation rather than a transcriptional regulation, which was believed to play an important role in DNMTI cytotoxic activities. [9] Taken together, it remains to be defined how many downstream effectors DNMTIs may activate or repress to achieve their anti-cancer efficacy. Moreover, little has been known whether there exist factors interfering with DNMTI's activity or conferring DNMTI resistance to malignant cells.

Human telomeres are nucleoprotein complex consisting of up to 15 kb TTAGGG repeat sequences and associated proteins or shelterin proteins including TRF1, TRF2, TPP1, POT1, TIN2 and RAP1. [10] Telomeres form protective caps on chromosome ends and are essential to genomic stability/integrity. [10] Progressive attrition of telomeric DNA occurs in normal somatic cells with each round of division and eventually cellular telomere becomes too short (dysfunctional) to exert a capping function. [10] Telomerase is an RNA-dependent DNA polymerase responsible for elongating telomere and absent in most somatic cells, consistent with telomere shortening in these cells. In sharp contrast, telomerase is widely activated in human malignancies including AML. [10–12] Activation of telomerase has been shown to be an essential step during oncogenesis, thereby stabilizing telomere length and conferring transformed cells unlimited proliferation potential. [10–14] Telomerase reverse transcriptase (TERT) is a catalytic component of the telomerase complex and a key determinant for controlling telomerase activity. In addition to its canonical telomere elongation function, TERT or telomerase has many other biological activities. [15–21] For instance, TERT has been observed to enhance survival, chemo-resistance, invasion and metastasis of malignant cells independently of its telomere lengthening function. [13, 18, 22–24]

Given the key role of TERT and telomere stabilization in cancer development and progression, it is important to address the effect of DNMTIs on TERT expression and telomere function for their anti-cancer activity. Indeed, the unique impact of DNA methylation on TERT transcription and telomere structure has been documented. [11] Unlike other gene promoters where their methylation silences gene transcription or expression, the methylated TERT promoter at certain sites is associated with the gene de-repression and telomerase activation in malignant cells. [11, 25–30] Published data have revealed that the effect of DNMTIs on TERT expression in malignant cells varies dependent on cell types. [25, 31–36] Importantly, as TERT is involved in chemo- and radio-resistance of malignant cells, [22–24] it is critical to elucidate whether TERT is capable of protecting DNMTI-mediated apoptosis. Moreover, it is currently unclear whether DNMT inhibition affects telomere function in AML cells. The present study was designed to address all these issues.

## RESULTS

### AML cell growth arrest and apoptosis is induced by 5-AZA in time- and dose-dependent manners

**Figure 1:**
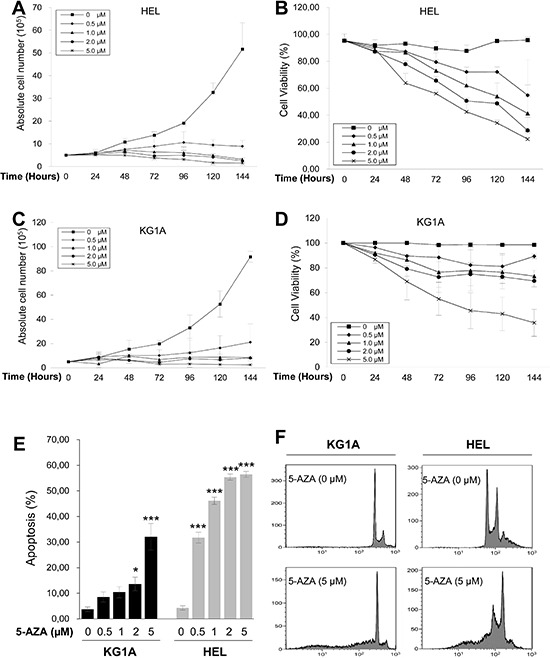
Growth arrest and apoptosis induction of AML cells by 5-AZA in a time and dose-dependent manner KG1A and HEL cells were treated with 5-AZA at different concentrations for up to 144 hours (6 days), and then analyzed for cell number, viability, cell cycle and apoptosis. **(A)** and **(B)** The number and viability of HEL cells in the presence of 5-AZA at various concentrations and time periods, respectively. **(C)** and **(D)** The number and viability of HEL cells in the presence of 5-AZA at various concentration and time periods, respectively. **(E)** Apoptosis of KG1A and HEL cells in the presence of 5-AZA at various concentrations. * and *** denote *P* < 0.05 and 0.001, respectively. **(F)** Representative FACS histograms showing PI staining of KG1A and HEL cells with and without 5-AZA. The values are means ± SD. Three independent experiments were performed.

We incubated AML HEL and KG1A cells with different concentrations of 5-AZA for various time periods, and then determined cell numbers and viability. Both HEL and KG1A cells proliferated exponentially when cultured in 5-AZA-free medium, while their exposure to 5-AZA attenuated or abolished increase in cell numbers (Figure 1). Compared to KG1A cells, HEL cells were more sensitive to 5-AZA and their proliferation almost completely stopped even at 0.5 μM (Figure 1A and 1C). The cell number decreased dramatically by day 6 (144 hrs) in the presence of 1.0 μM or more of 5-AZA (Figure 1A) and only 1.5 × 10^5^ HEL cells (less than 3% of untreated cells) were left at 5.0 μM of 5-AZA. The trypan blue exclusion test showed that the viability of HEL cells decreased progressively with increased amounts of 5-AZA and longer exposure (Figure 1B), and less than ¼ of cells survived at 5.0 μM by day 6. Similar results were obtained in 5-AZA-treated KG1A cells (Figure 1D). In addition, when other AML cells (Kasumi cell line) were incubated with 5-AZA, almost identical findings were produced (data not shown).

To see whether the low viability of 5-AZA-treated cells was due to apoptotic cell death, we performed Propidium iodide (PI) staining. Flow cytometry analyses revealed the sub-G1 cell accumulation of 5-AZA-treated cells in time- and dose-dependent manners (Figure 1E and 1F), demonstrating that 5-AZA induced apoptosis, consistent with the viability assay results in the same setting of cells.

### 5-AZA treatment leads to DNA damage and telomere dysfunction in AML cells

**Figure 2:**
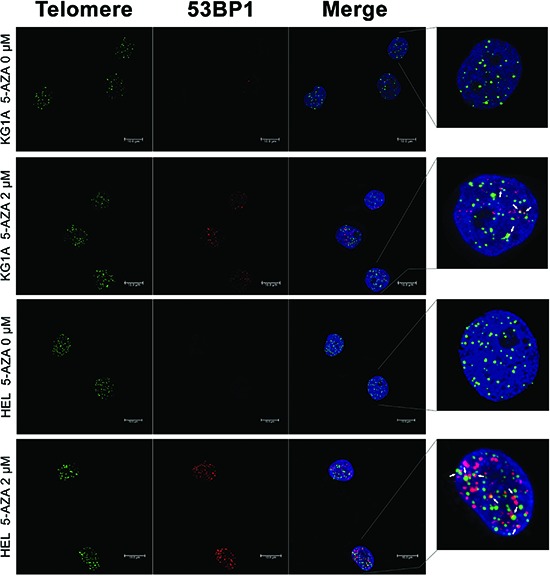
DNA damage and telomere dysfunction mediated by 5-AZA in AML cells KG1A and HEL cells were treated with 5-AZA at 2.0 μM for 72 hours and then analyzed for 53-BP1 foci and co-localization of telomere signals with 53-BP1 foci using Immuno-FISH. Red and Green: 53-BP1 foci and telomere signals, respectively. Yellow: Co-localization of 53-BP1 foci and telomere signals. Shown is the representative of three independent experiments.

A few of previously published studies indicate that 5-AZA-mediated cancer cell apoptosis is associated with DNA damage response. [37, 38] To see whether it occurs in 5-AZA-treated AML cells, we determined the focal formation of the checkpoint protein p53BP1, a well-established marker for DNA damage response, by using immunofluorescence (IF). 53BP1 foci were readily observed in 5-AZA-treated cells (Red, Figure 2), while rarely present in non-treated cells (Figure 2). These results clearly showed that DNA damage response was induced by 5-AZA in KG1A and HEL AML cells.

We further asked whether 5-AZA treatment led to telomere dysfunction. For this purpose, we examined the presence of dysfunctional telomere-induced foci (TIF): co-localization of 53BP1 foci with telomere signals using immuno-fluorescence in situ hybridization (Immuno-FISH). As shown in Figure 2, telomeres, revealed as green signals, were readily detectable in both control and 5-AZA-treated KG1A and HEL cells, whereas red 53BP1 foci only occurred in the treated cells. The merged image demonstrated that parts of 53BP1 foci were localized at telomeres in cells exposed to 5-AZA (TIFs: 3.60 ± 2.16/cell) while rarely seen in non-treated cells. It is evident from these results that 5-AZA induces telomere dysfunction (Figure 2).

### 5-AZA shortens telomere length in AML cells

**Figure 3:**
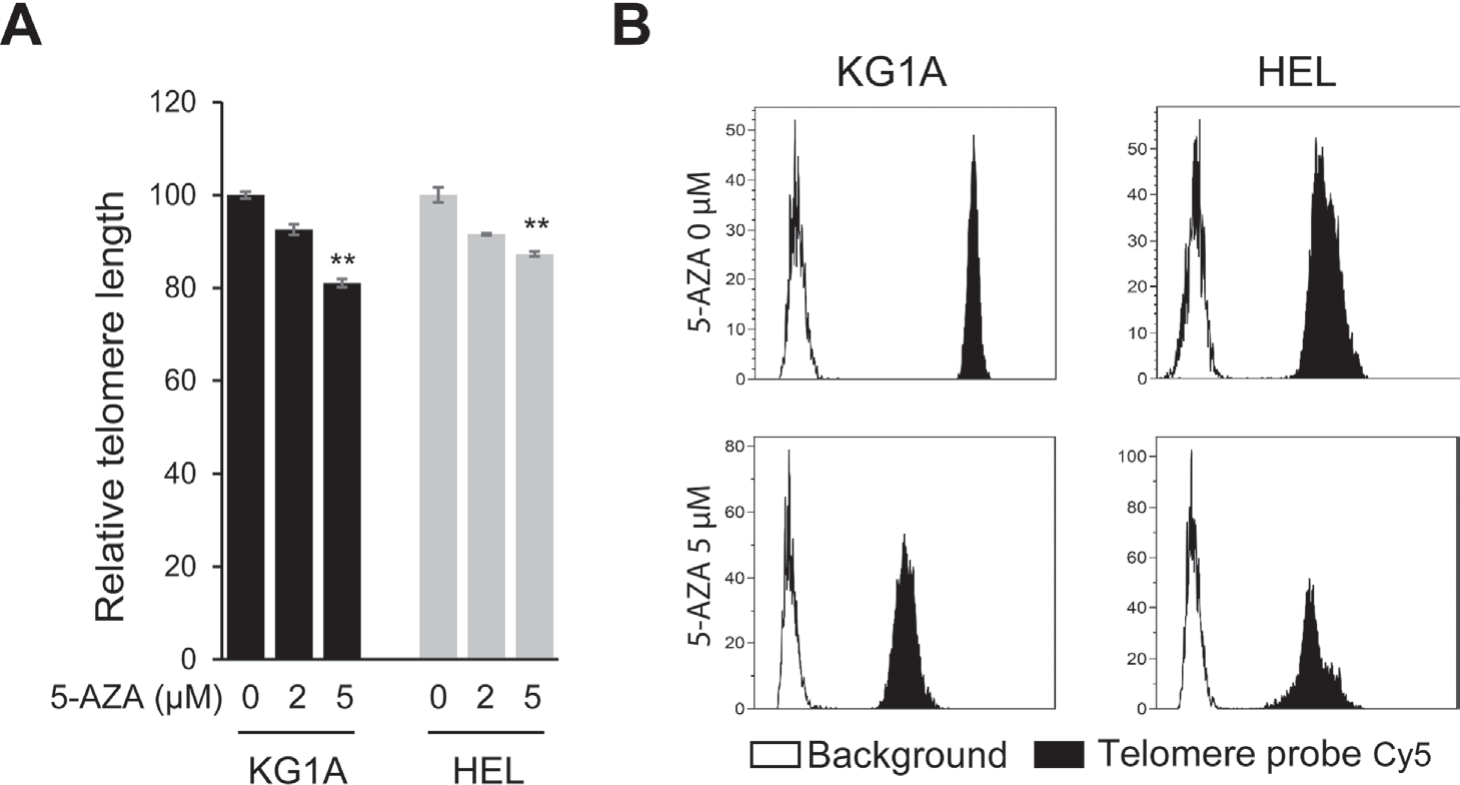
Telomere shortening in 5-AZA-treated AML cells **(A)** KG1A and HEL cells were treated with 5-AZA (2.0 and 5.0 μM, respectively) for 72 hours and telomere length was determined using FLOW-FISH. ** denotes *P* < 0.01. The values are means ± SD. **(B)** Shown are representative telomere signals as detected using FLOW-FISH. Three independent experiments were performed.

To probe potential mechanisms behind 5-AZA-mediated telomere dysfunction, we determined telomere length in those AML cells under study. Both KG1A and HEL cells were incubated with 2.0 and 5.0 μM of 5-AZA for 72 hours and then analyzed for telomere length using FLOW FISH analysis. Compared to the non-treated cells, both KG1A and HEL cells in the presence of 5-AZA at 2.5 μM only exhibited slight telomere shortening, however, significant telomere attrition was observed at 5.0 μM (Figure 3A and 3B).

### 5-AZA does not change the methylation of subtelomeric DNA

**Figure 4:**
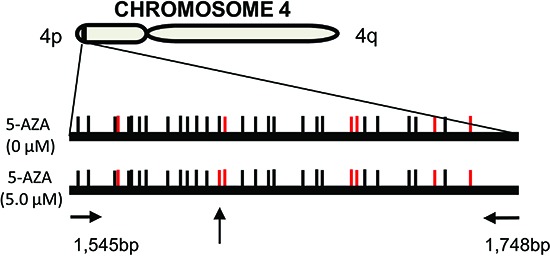
The methylation profile of subtelomeric DNA in 5-AZA-treated AML cells HEL cells were treated with 5.0 μM of 5-AZA for 72 hours and the methylation of subtelomeric DNA at chromosome 4p was assessed using methylation-specific PCR plus Sanger sequencing. CpGs are represented by vertical lines. Black and red lines indicate methylated and demethylated CpGs, respectively. The locations of the PCR primers are shown and the vertical arrow points to different methylation status of CpGs between control and 5-AZA-treated cells. Shown is the representative of two independent experiments.

It was previously shown that the chromatin structure of telomere and subtelomeric DNA affected telomere function, whereas the methylation status of subtelomeres substantially contributed to chromatin configuration locally. [39, 40] We thus examined alterations in subtelomere methylation profiles in HEL cells. Methylation-specific PCR was performed to amplify the subtelomeric region at chromosome 4p and amplicons were then analysed using Sanger sequencing (Figure 4). There were a total of 31 CpGs in the amplified region and 25 of them were methylated in untreated HEL cells (Figure 4). Twenty-four of the 25 methylated CpGs remained and only one of them became unmethylated in 5-AZA (5.0 μM) treated cells (Figure 4). These results suggest that the methylated CpGs at the subtelomeric DNA are resistant to DNMTIs.

### 5-AZA inhibits TERT expression in AML cells

**Figure 5:**
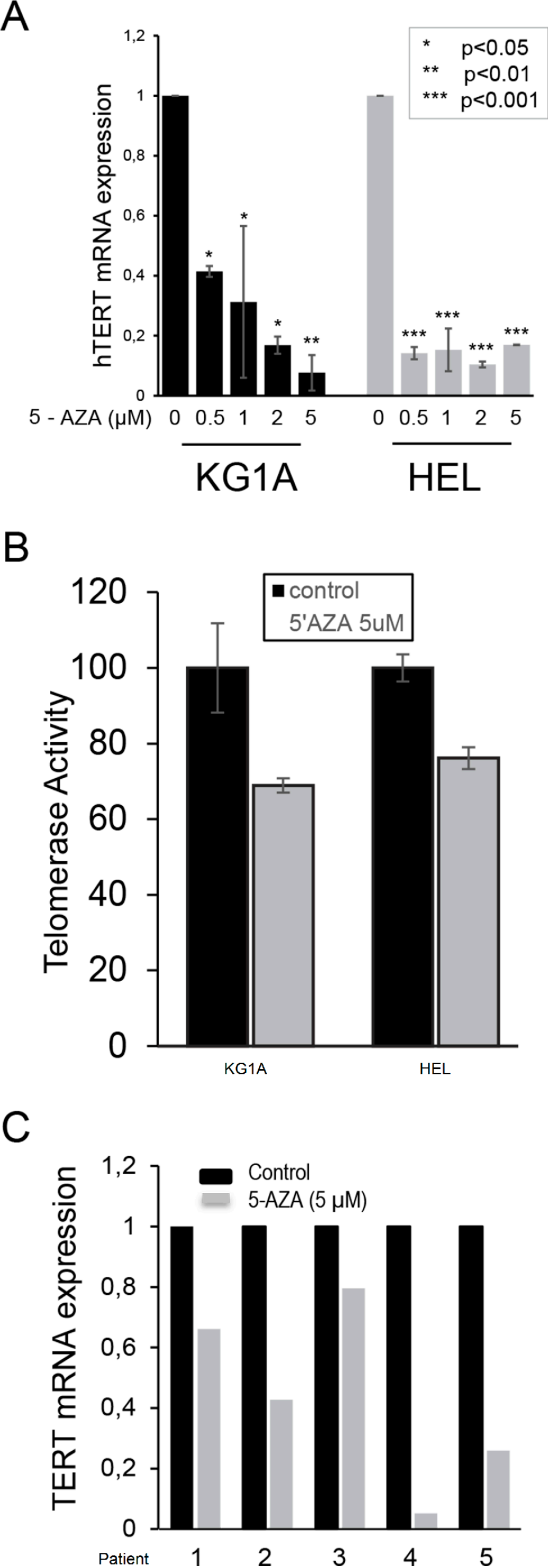
Down-regulation of TERT mRNA expression and telomerase activity in 5-AZA-treated AML cells KG1A and HEL cells were treated with 5-AZA for 72 hours, and TERT mRNA **(A)** and telomerase activity **(B)** were then determined using qPCR and Telomerase ELISA kit, respectively. Both TERT mRNA and telomerase activity in 5-AZA-treated cells were expressed as the percentage of those in control cells. * denotes *P* < 0.05. **(C)** The diminished TERT mRNA expression in 5-AZA-treated primary leukemic cells isolated from 5 patients with AML. Leukemic cells were treated with 5-AZA at 5.0 μM for 72 hours then analysed for TERT mRNA expression using qPCR. The level of TERT mRNA in the treated cells was expressed as that in control cells. The values are means ± SD. Three independent experiments were performed.

The effect of DNA methylation inhibition on TERT expression is dependent on the cell type under study. [25, 31, 32, 35, 36] To further explore the mechanism behind telomere shortening in 5-AZA-treated cells, we determined whether 5-AZA inhibited TERT expression and telomerase activity in KG1A and HEL cells. Cells were incubated with different concentrations of 5-AZA for 72 hours and then analyzed for their TERT mRNA level using quantitative PCR (qPCR). As shown in Figure 5A, TERT mRNA expression was significantly down-regulated by 5-AZA treatment in KG1A cells in a dose-dependent manner and remaining TERT transcripts were < 10% of control levels at 5.0 μM of 5-AZA (Figure 5A). Largely same results were obtained from HEL cells in the presence of 5-AZA (Figure 5A). Consistent with the down-regulation of TERT expression, telomerase activity was diminished in those 5-AZA-treated cells, but with less extent, probably due to a long half-life of the enzymatic activity (Figure 5B). [41]

To determine whether the results obtained from AML cell lines could be recapitulated in primary AML cells, we further tested the 5-AZA effect on primary leukemic cells derived from five newly diagnosed AML patients (Table 1). Cells were incubated with 5-AZA and TERT transcripts were then assessed. As shown in Figure 5C, 5-AZA treatment led to the down-regulation of TERT mRNA to different extents in leukemic cells from all five patients.

**Table 1:** Characteristics of five patients with acute myeloid leukemia

Patient	Gender	Age (years)	Diagnosis	Cytogenetics	Molecular Abnormalities
1	Female	22	AML-M3	t(15;17)	*FLT3-ITD* mutation
2	Male	20	AML-M4E0	inv16(p13q22)	*c-KIT* mutation
3	Male	60	AML-M5	Normal	
4	Male	78	AML	del(20)	*FLT3-ITD* mutation
5	Male	68	AML-M1	Normal	

### TERT over-expression attenuates telomere shortening, telomere dysfunction, DNA damage and apoptosis in 5-AZA-treated AML cells

**Figure 6:**
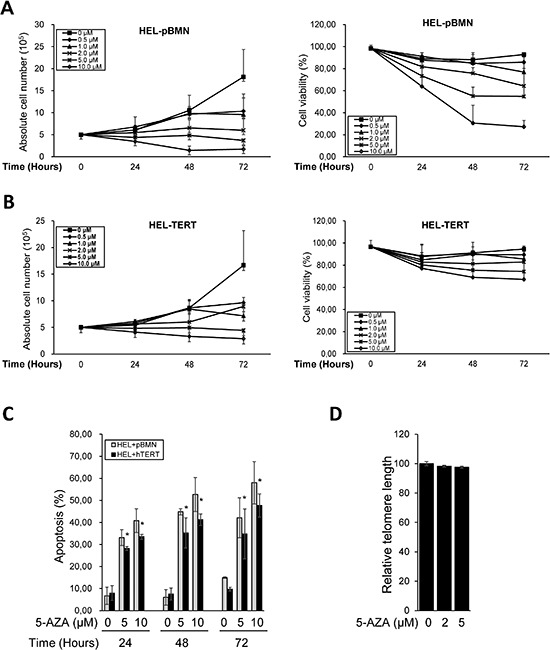
Attenuation of diminished viability, apoptosis, and telomere shortening by ectopic TERT expression in 5-AZA-treated HEL cells HEL-TERT and HEL-pBMN cells were treated with 5-AZA at different concentrations for up to 72 hours (3 days), and then analyzed for cell number, viability, and apoptosis and telomere length. **(A)** and **(B)** The number and viability of HEL-TERT and HEL-pBMN cells in the presence of 5-AZA at various concentrations and time periods, respectively. **(C)** Differences in apoptosis of HEL-TERT and HEL-pBMN cells in the presence of 5-AZA at various concentrations. * denotes *P* < 0.05. **(D)** Telomere length in 5-AZA-treated HEL-TERT cells as determined using FLOW-FISH. The values are means ± SD. Three independent experiments were performed.

**Figure 7:**
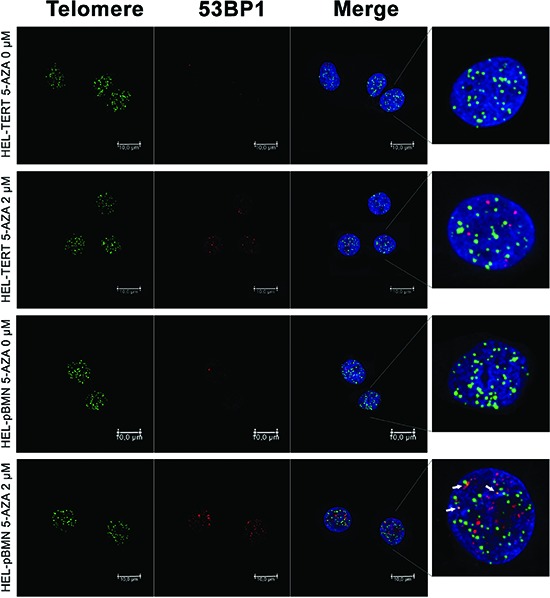
Attenuation of telomere dysfunction and DNA damage by ectopic TERT expression in 5-AZA-treated HEL cells HEL-TERT and HEL-pBMN cells were treated with 5-AZA at 2.0 μM for 72 hours and Immuno-FISH was performed to determine 53-BP1 foci and telomere-dysfunctional foci (TIF). Red and Green: 53-BP1 foci and telomere signals, respectively. Yellow: Co-localization of 53-BP1 foci and telomere signals. Shown is the representative of three independent experiments.

Given all the above observations, we sought to determine whether ectopic TERT expression was capable of antagonizing the effect of 5-AZA. For this purpose, we introduced a lenti-viral TERT expression vector into HEL cells, making a TERT-over-expressed HEL cell subline (HEL-TERT) (Figure 6A). This subline expressed two-fold higher telomerase activity than its parent one and TERT expression/telomerase activity was not inhibited by 5-AZA (data not shown). The parent control HEL cells with an empty pBMN vector (HEL-pBMN) and HEL-TERT cells were first treated with 5-AZA and potential differences in cell numbers, viability or apoptosis, telomere length, telomere dysfunction, DNA damage response were then compared between these two sublines. First, more HEL-TERT cells survived than HEL-pBMN cells in the presence of 5-AZA, especially at a high concentration (mean ± SD, 69.7 ± 7.9% vs 30.5 ± 16.2%, *P* = 0.021) (Figure 6A and 6B). Consistently, apoptotic death of HEL-TERT cells was 10% less than that of HEL-pBMN cells (*P* < 0.05) (Figure 6C). Second, there were no detectable decline in telomere length in HEL-TERT cells treated with 5-AZA at 5.0 μM (Figure 6D), which is in contrast to the results observed in parent HEL cells (Figure 3); Third, 5-AZA-induced TIFs and p53-BP1 foci were significantly fewer in HEL-TERT cells than in HEL-pBMN cells (TIFs: 2.20 ± 2.08/cell in HEL-TERT cells vs 3.60 ± 2.16/cell in HEL-pBMN cells, *P* = 0.019, Figure 7).

## DISCUSSION

DNMTIs have been used for the treatment of certain hematological malignancies including AML, but the underlying mechanism of anti-tumor activity remains incompletely understood. The findings presented here demonstrate that 5-AZA inhibited TERT expression in AML cells, shortened telomere and induced telomere dysfunction that subsequently results in apoptotic death of AML cells. Ectopic TERT expression partially attenuated telomere dysfunction and apoptosis. Thus, telomere dysfunction mediated by 5-AZA may contribute to the anti-tumor activity of DNMTIs.

The gene promoter methylation status is intimately associated with gene transcription. In general, promoter hyper-methylation represses while hypo-methylation stimulates expression of given genes. However, the TERT promoter is very unique: widespread DNA demethylation of the promoter occurs in human fibroblasts and other normal cells where the TERT transcription is stringently repressed, whereas the hyper-methylation of the specific TERT promoter region is required for the gene activation in many kinds of malignant cells. [25–30] Therefore, the effect of DNMTIs on TERT expression is dependent on cell type and context. Our present results show that 5-AZA treatment led to down-regulation of TERT expression coupled with diminished telomerase activity in KG1A and HEL cells, indicating the methylation-dependent TERT regulation in AML cells. It is currently unclear how exactly 5-AZA controls TERT expression. Kitagawa et al [31] showed that the DNMTI-mediated TERT down-regulation resulted from diminished expression of c-MYC, a key trans-activator of the *TERT* gene. It has also been proposed that the specific methylation at the TERT promoter region prevents putative repressors from binding to the promoter, thereby de-repressing gene transcription.

Sufficient telomere length and appropriate levels of telomere binding factors or shelterin proteins are required for maintenance of telomere function. [10] Based on our present results, 5-AZA likely leads to telomere dysfunction via telomere attrition. We observed that TERT mRNA down-regulation was coupled with a decline in telomerase activity in 5-AZA-treated AML cells, which consequently disrupts telomere length stabilization, and triggers telomere dysfunction. Indeed, telomere shortening was observed in AML cells incubated with 5-AZA. Moreover, TERT over-expression prevented 5-AZA-mediated telomere shortening in those cells. Despite these supportive observations, however, TERT down-regulation could not be the only explanation for telomere shortening seen in 5-AZA-treated cells, because numerous studies have demonstrated that there is a significant time-lag between telomere shortening and cancer cell exposure to either telomerase inhibitors or TERT inhibition. [42] In addition, our findings are in contrast to those of previous studies showing that DNMT inhibition promoted telomere elongation in mouse and human cells. [39, 43] It is currently unclear what causes such a discrepancy between those studies. Likely, cells derived from different species and the use of different tissue systems contribute to the varying response to DNMT inhibition.

Alterations in chromatin structure are closely associated with telomere structure and function. Gonzalo et al found that DNMT depletion resulted in demethylation of subtelomeric regions. [39, 40] We thus hypothesized that the 5-AZA treatment might lead to the hypomethylation of the subtelomere region in leukemic cells in the presence of 5-AZA. Unexpectedly, however, the methylation profile in the subtelomere of chromosome 4p did not differ substantially between the cells with and without 5-AZA treatment. The implications of these seemingly contradictory results remain to be resolved. Again, cell origin, differing culture conditions and different approaches to DNMT inhibition might contribute to the observed discrepancy. Further analyses of the global DNA methylation profile in non-treated and DNMTi-treated cells are required to elucidate these issues.

DNA damage response was previously observed in DNMTI-treated leukemic and cancer cells. [8, 37, 38, 44, 45] DNMTIs were shown to cause replication lesions through which double-strand breaks of DNA, radial chromosomes and chromatid breaks are induced. [44] Jiemjit et al found that DNA damage also occurred in DNMT−/− cells exposed to DNMTIs, indicating that such effect is DNMT inhibition-independent. [37] It is currently unclear how exactly DNMTIs trigger DNA damage. Given the fact that DNA damage response is required for growth arrest and apoptosis mediated by DNMTIs, [37] it is a demanding task to elucidate the underlying mechanism. Moreover, another key issue is whether the same DNA damage mechanism contributes to telomere dysfunction. Because telomeric DNA damage is irreparable, [46] cells carrying telomere dysfunction will eventually undergo apoptosis or senescence, which favours eradication of malignant cells and has an important therapeutic implications.

TERT has been shown to possess multiple non-canonical activities in addition to its telomere lengthening function. [13, 15–21, 47, 48] For instance, TERT is capable of facilitating the recruitment of DNA repair factors to sites of double-stranded breaks, [48] while depletion of TERT leads to enhanced cell radiosensitivity, and diminished capacity for DNA repair. [47] In accordance with these reports, we observed that TERT over-expression attenuated DNA damage, as demonstrated by decreased 53-BP1 foci in 5-AZA-treated leukemic cells. Because DNA damage is required for growth arrest and apoptosis mediated by DNMTIs, [37] it is not surprising that cell death was attenuated in TERT over-expressed cells when exposed to 5-AZA, likely due to accelerated DNA repair mediated by TERT.

In the present study, we also observed that 5-AZA inhibited TERT expression in primary leukemic cells derived from all five AML patients. We were unable to determine their telomere length and function, DNA damage and apoptosis due to limited cell numbers. For the same reason, the relationship between the sensitivity of leukemic cells to 5-AZA and the degree of TERT down-regulation could not be assessed in these primary AML cells. These issues are clinically important and should be addressed in further studies.

In summary, we show that the DNMT inhibitor 5-AZA down-regulates TERT expression in both AML cell lines and primary leukemic cells, and shortens telomere length coupled with telomere dysfunction, DNA damage response and apoptosis. Ectopic TERT expression partially attenuated telomere dysfunction and DNA damage, thereby protecting AML cells from apoptosis. Conceivably, TERT down-regulation and telomere dysfunction mediated by 5-AZA may contribute to the anti-tumor activity of DNMTIs. Thus, it may be worthwhile to evaluate the therapeutic efficacy of DNMTIs on hematological malignancies based on their induction of TERT inhibition/telomere dysfunction in future clinical trials.

## MATERIALS AND METHODS

### Cells, cell cultures and reagents

The study included the human AML cell lines KG1A and HEL that were grown in 10% foetal calf serum-containing RPMI-1640 with addition of 2 mM L-glutamine and antibiotics (50 mg/mL penicillin, and 50 mg/mL streptomycin) in a humid atmosphere at 37°C/5% CO_2_. The DNA methylation inhibitor 5-AZA was bought from Sigma-Aldrich (St. Louis, USA) and exponentially growing cells were cultured in the presence and absence of 5-AZA (0, 0.5, 1, 2 and 5 μM) for up to 144 hours or 6 days. Culture medium was replaced with freshly prepared 5-AZA-containing medium every two days. Cells were counted for numbers and viability determined by using trypan Blue exclusion test.

### Primary AML cell separation and culture

Primary AML cells were derived from five AML patients and their clinical/molecular characteristics are listed in Table 1. Peripheral blood was drawn into heparinized glass tubes and leukemic cells were isolated by Lymphoprep gradient centrifugation (Nycomed, Oslo, Norway), and subsequently incubated in complete medium in the absence or presence of 5-AZA as described above. The study was approved by the Karolinska Ethics Review Committee.

### The TERT lenti-viral vector and infection of AML cells

A lenti-III-HA-GFP-TERT vector was constructed and a Lenti-BMN-GFP vector (a gift from Rudbeck Laboratory, Department of Immunology, Genetics and Pathology of Uppsala University) was used as control. The lentiviral vector was packaged in 293FT cells and supernatant collected to infect HEL cells. The cells were selected using puromycin (2 μg/ml).

### RNA extraction, reverse transcription and qPCR

Total cellular RNA in cells with different treatments was extracted using Trizol (Life Technology, Paisley, Scotland, UK). cDNA was synthesized using random primers (N6) (Amersham, Buckinghamshire, UK) and M-MLV reverse transcriptase. qPCR was carried out in an ABI7700 sequence detector (Applied Biosystems, Foster City, CA, USA) using SYBR Green kit (Applied Biosystems, Foster City, CA) and the specific primer pair for TERT transcripts, as previously described. [49] β2-microglobulin (β2-M) was PCR-amplified as an internal control. Levels of TERTmRNA were calculated based on the threshold values and normalization of β2-M expression.

### Assessment of telomerase activity

Telomerase activity was assessed using a commercial Telomerase PCR ELISA kit (Roche Diagnostics Scandinavia AB, Stockholm, Sweden) as recommended by the manufacturer. For each assay, one μg of protein was used, and 25 PCR cycles were performed after the telomerase-primer elongation reaction. The PCR products were detected using ELISA color reaction and the level of telomerase activity was expressed as absorbance in arbitrary units.

### Flow cytometry analysis of cell cycle and apoptosis

AML cells were treated with different concentrations of 5-Azacytidine for three consecutive days as decribed above, and then harvested for ethanol fixation and PI staining. The PI fluorescence was measured with a FlowCytometer (Beckman Coulter). For each sample 1 × 10^6^ cells were measured. Data analysis was permormed with Kaluza®Flow Analysis Software. The control gate was set based on the negative control.

### Immuno-FISH

Immuno-FISH was performed as described. [50] Briefly, cells were harvested and cytospined onto Superfrost plus slides (Thermo Scientific), fixed with 4% paraformadehyde and permeabilized with Triton PBS for 20 mins and blocked with serum free Block (DAKO, Glostrup, Denmark). The slides were then incubated with 53BP1 antibody (Bethyl Inc., Montgomery, Texas, USA) followed by incubation with Alexa 594 secondary antibody (Jackson Labs Technologies Inc., Los Gatos, CA, USA). The slides were treated with frozen and thawed cycle in liquid nitrogen, and incubated in 0.1N HCL for 10 mins. The PNA-telomere probe (PANAGENE Inc., Daejeon, Korea) was finally added. High-resolution images were collected using Leica Confocal TCS SP5 with 488 nm and 594 nm sequential laser scan. Depth of Z stack was taken with recommended optimization and between-stack mode. The co-localization of 53BP1 and telomere signals was examined and analyzed on each layer by two separate independent persons in a double-blind manner. The detailed protocol is available upon request.

### Flow-FISH for telomere length assay

Flow FISH of AML cells was performed according to previous protocols by Baerlocher et al [51, 52] with minor modifications. Cells from calf thymus were kindly donated from the butchery Ö-slakt AB (Värmdö, Stockholm). All experiments were made with a Gallios flow cytometer (Beckman Coulter) and analyzed using the Kaluza software (Beckman Coulter, Caguas, PR, USA). For quantification fluorescent MESF-FITC beads (Bangs Laboratories, Fishers, IN, USA) were used and the fluorescent signal was quantified using the QuickCal v.2.3 data analysis program (Bangs Laboratories).

### Subtelomeric DNA methylation at chromosome 4p

Genomic DNA, extracted from control and TERT-over-expressed HEL cells with or without 5-AZA, was subject to bisulfite conversion using an EZ DNA Methylation-Gold Kit (ZYMO RESEARCH, Irvine, CA, USA). PCR primers specific to the subtelomere region of chromosome 4p was used to amplify the target region. [53] The obtained PCR products were then sequenced at both directions. Two independent experiments were performed.

### Statistics

Student's T-test was used to compare cell numbers, apoptotic cells, TERT mRNA levels and telomere lengths between control and 5-AZA-treated AML cells. All the tests were two-tailed and computed using SigmaStat3.1® software (Systat Software, Inc., Richmond, CA, USA). *P* values of < 0.05 were regarded as statistically significant.
